# High quality transparent conductive Ag-based barium stannate multilayer flexible thin films

**DOI:** 10.1038/s41598-017-00178-9

**Published:** 2017-03-07

**Authors:** Muying Wu, Shihui Yu, Lin He, Lei Yang, Weifeng Zhang

**Affiliations:** 1 School of Electronic Engineering, Dongguan University of Technology, Guangdong, Dongguan 523808 China; 2 School of Electronic and Information Engineering, Tianjin University, Tianjin, 300072 P. R. China; 3 Key Laboratory of Photovoltaic Materials of Henan Province and School of Physics and Electronics, Henan University, Kaifeng, 475004 P. R. China

## Abstract

Transparent conductive multilayer thin films of silver (Ag)-embedded barium stannate (BaSnO_3_) structures have been deposited onto flexible polycarbonate substrates by magnetron sputtering at room temperature to develop an indium free transparent flexible electrode. The effect of thicknesses of Ag mid-layer and barium stannate layers on optical and electrical properties were investigated, and the mechanisms of conduction and transmittance were discussed. The highest value of figure of merit is 25.5 × 10^−3^ Ω^−1^ for the BaSnO_3_/Ag/BaSnO_3_ multilayer flexible thin films with 9 nm thick silver mid-layer and 50 nm thick barium stannate layers, while the average optical transmittance in the visible range from 380 to 780 nm is above 87%, the resistivity is 9.66 × 10^−5^ Ω · cm, and the sheet resistance is 9.89 Ω/sq. The change rate of resistivity is under 10% after repeated bending of the multilayer flexible thin films. These results indicate that Ag-based barium stannate multilayer flexible thin films can be used as transparent flexible electrodes in various flexible optoelectronic devices.

## Introduction

Flexible and transparent electronics, such as transparent flexible electronic circuits, organic photovoltaics, flexible electronic book, flat panels wearable computers and flexible organic light emitting diodes^1, 2^, are expected to meet emerging technological demands where silicon based electronics cannot provide a solution^3–5^. The key components of flexible and transparent electronics is the flexible transparent conductive thin films. So far, the flexible thin films of transparent conductive indium tin oxide (ITO) have been investigated extensively and applications due to its low resistivity (≤10^−3^ Ω · cm) and high light transmittance (≥80%) in the visible region^6, 7^. However, indium is very costly (almost US $1400/kg), driven by its scarcity^8^. Thus, it is important to develop cheaper materials with good opto-electrical properties.

Some potential alternative materials such as doped zinc oxide (ZnO) and doped tin oxide (SnO_2_) have been suggested as promising alternatives to flexible transparent conductive ITO thin films^9–11^. That, unfortunately, the resistivity is still not low enough for practical applications. Fabricating high-performance flexible transparent conductive thin films is challenging owing to a trade-off between processing temperature and film performance. Recently, some researchers have tried to solve this problem by using a novel structure—a sandwich structure of dielectric/metal/dielectric multilayer system^12–14^. This structure have flexibility, low resistivity and high transparence due to the reflection from the metal layer can be suppressed by the multilayer system and obtain a higher transparent effect^15^. Amongst metals, a practical use are gold (Au), silver (Ag) and cuprum (Cu) because of their low resistivity. However, compared with Ag, Al is more sensitive to oxygen, and Au is more expensive. Therefore, we select Ag as the metal layer.

Barium stannate (BaSnO_3_, BS) is a potential semiconductor material and find a variety of applications in modern technology in different components ranging from optoelectronics, thermally stable capacitors, gas sensors, humidity sensors, etc^15–17^. BaSnO_3_ as an n-type semiconductor with wide band gap (3.4 eV), high carrier mobility, high transparency (>90%) in the wavelength range from 380 nm to 2600 nm, and good adhesion to the glass and plastic substrates^16, 17^. Apart from this, it is chemically and thermally stable under hydrogen plasma processes that can be used as optical coatings for the production of flexible and transparent electronics.

Among the processes used to prepare transparent conductive thin films, magnetron sputtering is considered to be the most favorable deposition method, because of its low cost, good adhesion, high deposition rate, environmentally friendly, uniform thickness over large areas, and easily controlled deposition process^18, 19^. In this paper, a sandwich structure of BaSnO_3_/Ag/BaSnO_3_ multilayer flexible transparent conductive thin film system was designed and deposited onto polycarbonate (PC) flexible substrates by RF and DC magnetrons sputtering. The thicknesses of BaSnO_3_ and Ag layers were used as the design parameters in optimization process. The performance of BAB multilayer thin films based on the optical and electrical properties has been evaluated using a figure of merit. The conduction mechanism as a function of Ag layer thickness and the role of Ag layer on the transmission properties are investigated. The bend properties of multilayer flexible thin films are studied as well.

## Results

### Effect of the Ag mid-layer thickness

**Figure 1:**
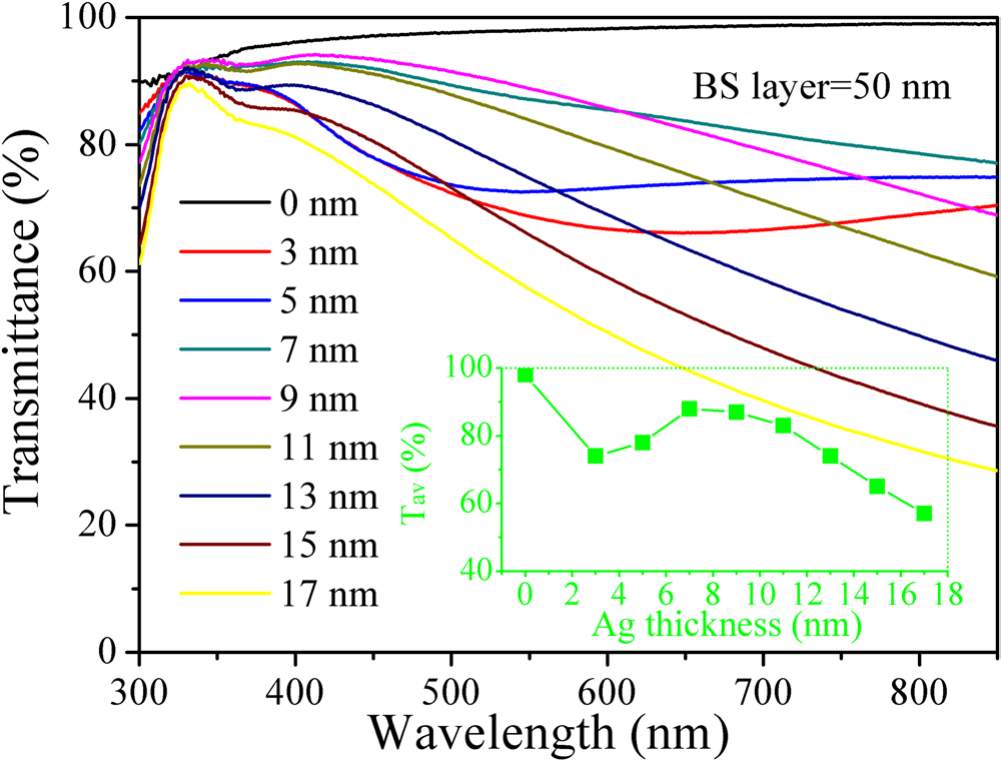
Transmittance spectra of BS/Ag/BS multilayer flexible thin films with various Ag mid–layer thicknesses. Inset is the average optical transmittance in the visible range (380 nm~780 nm) of the multilayer flexible thin films as a function of Ag mid-layer thickness.

The X–ray diffraction (XRD) and AFM results for BS/Ag/BS multilayer flexible thin films with different thicknesses of the middle Ag layer were shown in Figs S1 and S2 of the Supplementary Information (SI). The optical transmittance of BS/Ag/BS multilayer flexible thin films deposited at room temperature in the wavelength range 300–800 nm, as a function of Ag thickness are shown in Fig. 1. The average optical transmittance in the visible range (380–780 nm) can be determined as follows^20^:

1 $${T}_{av}=\frac{\int V(\lambda )T(\lambda )d\lambda }{\int V(\lambda )d\lambda }$$

where *T*_*av*_ is the average optical transmittance in the visible range, *V*(*λ*) is the luminous spectral efficiency function defining the standard observer for photometry^20^ and *T*(*λ*) is the measured transmittance of BS/Ag/BS multilayer flexible thin films. The inset shows the average optical transmittance in the visible range of the multilayer flexible thin films with various Ag thicknesses. It is seen that the average transmittance of bare BS thin films are ~98%. After insertion of the Ag layer, the average transmittance of the multilayer flexible thin films with 3 nm thick Ag mid-layer drops to 74%. With increasing Ag thickness, there is a parabolic increase in average optical transmittance. When the thickness of Ag mid-layer is 7 nm, the average transmittance has a remarkable increase, reaches a maximum of approximately 88%. However, the average transmittance decreases from ~88% to ~57% for the multilayer flexible thin films with 17 nm Ag mid-layer thickness with a further increase in Ag mid-layer thickness.

At a lower Ag mid-layer thickness of 5 nm, the Ag mid-layer is discontinuous, small Ag islands are formed on the bottom BS layer. Due to the scattering of light from the interface of BS/Ag in isolated Ag islands, the average transmittance (<80%) is fairly low. And, the light absorption of the aggregated Ag islands is also lead to the low transmittance. For the dielectric/metal/dielectric multilayer thin films, the optical absorption is strongly affected by the surface plasmonic effect^21^. The planer surface plasmonic coupling is non-radiative, resulting in high optical absorption of BS/Ag/BS multilayer flexible thin films^21, 22^. The propagation length of surface plasmonic coupling mode can be derived from the following equation^21^:

2 $${\delta }_{sp}=\frac{c}{\omega }{(\frac{{\varepsilon }_{m}^{^{\prime} }}{{\varepsilon }_{d}}+\frac{{\varepsilon }_{d}}{{\varepsilon }_{m}^{^{\prime} }})}^{3/2}\frac{({\varepsilon }_{m}^{^{\prime} })}{{\varepsilon }_{m}^{^{\prime\prime} }}$$

Where *δ*_*sp*_ is the propagation length of surface plasmonic coupling mode, *ε*_*d*_ is the dielectric constant of BS layer, ${\varepsilon }_{m}^{^{\prime} }$ and ${\varepsilon }_{m}^{^{\prime\prime} }$ are the real and imaginary parts of the dielectric function of the silver. From the Eq. (2), the propagation length decreases for the structures with increase in imaginary parts of the dielectric function of the silver. When the Ag layer is discontinuous, the limitation of the mean free path of the conduction electrons leads to the existence of a size-dependent variation in the dielectric function^23^. For this reason, the imaginary part of the overall dielectric function $({\varepsilon }_{m}^{^{\prime\prime} })$ of the silver can be corrected as^23, 24^

3 $${\varepsilon }_{m}^{^{\prime\prime} }{\varepsilon }_{m}^{^{\prime} }+\frac{D}{R}$$

where *D* represents a constant at a particular wavelength and *R* represents the particle radius. According to Eq. (3), the imaginary part of the overall dielectric function of the silver increases with decreases of Ag island size. As a result, the surface plasmonic coupling is suppressed, and the average transmittance decreases with smaller island size below the critical thickness. When the Ag mid-layer is at the critical thickness of 7 nm, the relative high transmittance can be attributed to the Ag becomes a continuous layer that causes decrease in light scattering. However, as further increasing the Ag thickness above the 7 nm, the average transmittance decreases, which is attributed to the increase in plasmon absorption and reflectivity of the mid Ag layer^9, 25^. As can be seen in Fig. 1 as well, there is a shift in the absorption edge to lower energies for the BS/Ag/BS multilayer flexible thin films with the increase of Ag mid-layer. The same trend was also observed by Alford group^26^ and Yu group^27^. For the multilayer flexible thin films, silver atoms in the Ag mid-layer may ionize partly and become positively charged. There is an electric field directed from the positively charged silver atoms to electrons in the conduction band of the BS layer, which would cause downward shifting of the conduction band and upward shifting of the valence band, and then resulting in the band gap shrinking, therefore, the absorption edge shows red shift phenomenon.

**Figure 2:**
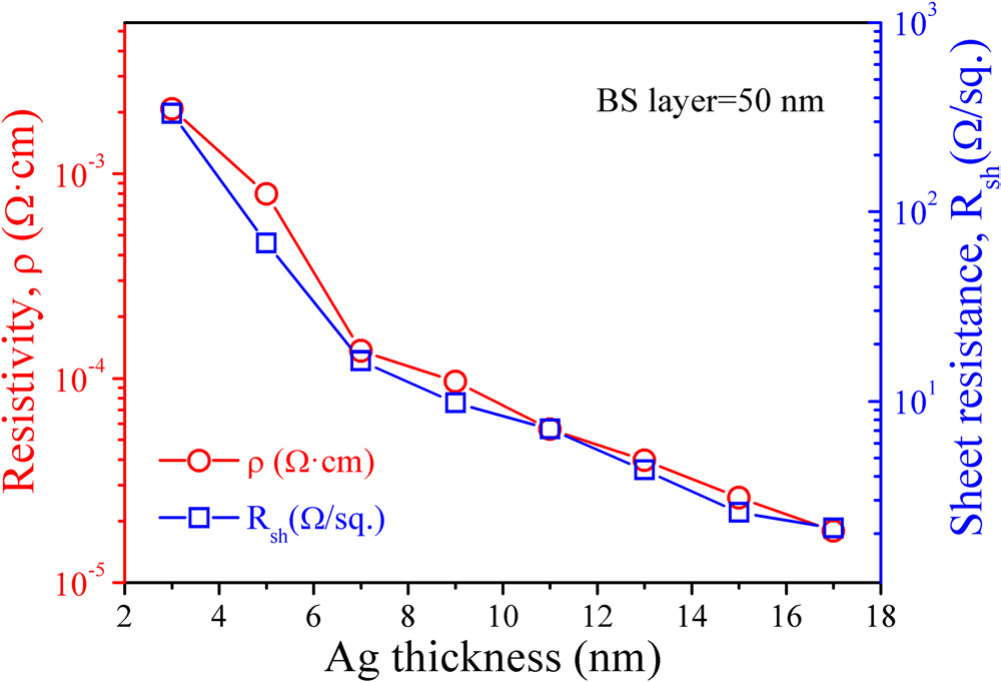
The dependence of resistivity and sheet resistance for BS/Ag/BS multilayer flexible thin films on the Ag mid–layer thickness. Inset is the schematic diagram of parallel resistors in multilayer flexible thin films.

Figure 2 shows the resistivity and sheet resistance of the BS/Ag/BS multilayer flexible thin films deposited at room temperature as a function of Ag mid-layer thickness. BaSnO_3_ is an insulator with resistivity of the order 10^6^ Ω · cm^16^. After insertion of the 3 nm thick Ag layer between the BS layers, the resistivity decreases drastically to 2.08 × 10^−3^ Ω · cm, which suggests that there is a nine orders of magnitude decrease in resistivity for the BS/Ag/BS multilayer flexible thin films when compared to bare BaSnO_3_. With increasing the Ag mid–layer thickness to 17 nm, the resistivity reaches to the minimum value of 1.79 × 10^−5^ Ω · cm. The resistivity (*ρ*) of the thin films is approximately proportional to its sheet resistance (*R*_*sh*_) (*ρ* ≈ *R*_*sh*_ *d*, where *d* is the thickness of multilayer flexible thin films)^27^, therefore, the sheet resistance curve follows similar trend to that resistivity as the increase of Ag mid-layer thickness (as shown in Fig. 2). The sheet resistance of BS/Ag/BS is a result of parallel combination of the three individual layers (as shown in inset of Fig. 2). The relationship among the sheet resistance of the BS/Ag/BS multilayer flexible thin films (*R*_*sh*_), BaSnO_3_ layer (*R*_*BS*_), and Ag mid-layer (*R*_Ag_) is expressed as followed:

4 $$\frac{1}{{R}_{sh}}=\frac{2}{{R}_{{\rm{BS}}}}+\frac{1}{{R}_{{\rm{Ag}}}}$$

So

5 $$\rho =d{(\frac{{d}_{{\rm{Ag}}}}{{\rho }_{{\rm{Ag}}}}+2\frac{{d}_{BS}}{{\rho }_{{\rm{BS}}}})}^{-1}$$

Where, *ρ*_*Ag*_, *ρ*_*BS*_, *d*_Ag_ and *d*_*BS*_ are the resistivity of Ag mid-layer, the resistivity of BS layer, the thickness of Ag mid-layer and the thickness of BS layer, respectively. Since *ρ*_*Ag*_ ≪ *ρ*_*BS*_, the resistance of multilayer flexible thin films depends mainly on the Ag layer.

For 3 nm Ag mid-layer thickness, the silver islands are small and separated by the BS dielectric, and the distance between islands is large, the conduction through the BS dielectric main is caused by thermionic emission or carrier injection^28^, therefore, the resistivity is large. As the thickness of Ag increases, the Ag islands grow, some of the islands undergo large scale coalescence^29^, the separation between the islands reduces, and a process of activated tunneling of electrons occurs, which leads to a remarkably decrease in resistivity. The conductivity (*ρ*) for the thickness of Ag below a critical value (7 nm), is governed according to the following equation^30^:

6 $$\rho \propto \exp (-2\beta \upsilon -\frac{W}{kT})$$

where *υ* represents the island separation, *W* represents the island charging energy, *β* represents the tunneling exponent of electron wave functions in the insulator, *k* represents the Boltzmann constant, and *T* is the temperature. From the Eq. (6), the separation between the Ag islands and the island size govern the conductivity. That is to say, as an increase in the thickness of Ag mid-layer, the decrease of resistivity is due to the decrease of the separation between Ag islands. When the thickness of Ag mid-layer is above the critical value (7 nm), the spaces between the Ag islands are filled, the Ag islands form a near-contiguous layer with many holes and cracks^31^. Therefore, the decrease in resistivity is governed by the combined effect of the increase in carrier concentration of conducting electrons and mobility. In the Ag thickness regime above 7 nm, the conductivity was governed by the following equation^30^:

7 $$\frac{\rho }{{\rho }_{0}}\propto \frac{3}{4}(1-p)\kappa \,\mathrm{log}\,\frac{1}{\kappa }$$

8 $$\kappa =\frac{{d}_{Ag}}{{\lambda }_{0}}$$

where *ρ*_*0*_ represents the resistivity of the bulk silver, *p* represents the fraction of the distribution function of the electrons arriving at the surface, and *λ*_0_ represents the mean free path of the conducting electrons. In this regime, the thickness of Ag layer approaches the mean free path of the conducting electrons, the resistivity is mainly influenced the surface scattering. As increasing silver layer thickness, the holes decrease in the surface of Ag layer, and the interface regions became a smaller fraction of the total thickness. Therefore, the resistivity gradually decreased to nearly the value of bulk silver.

**Figure 3:**
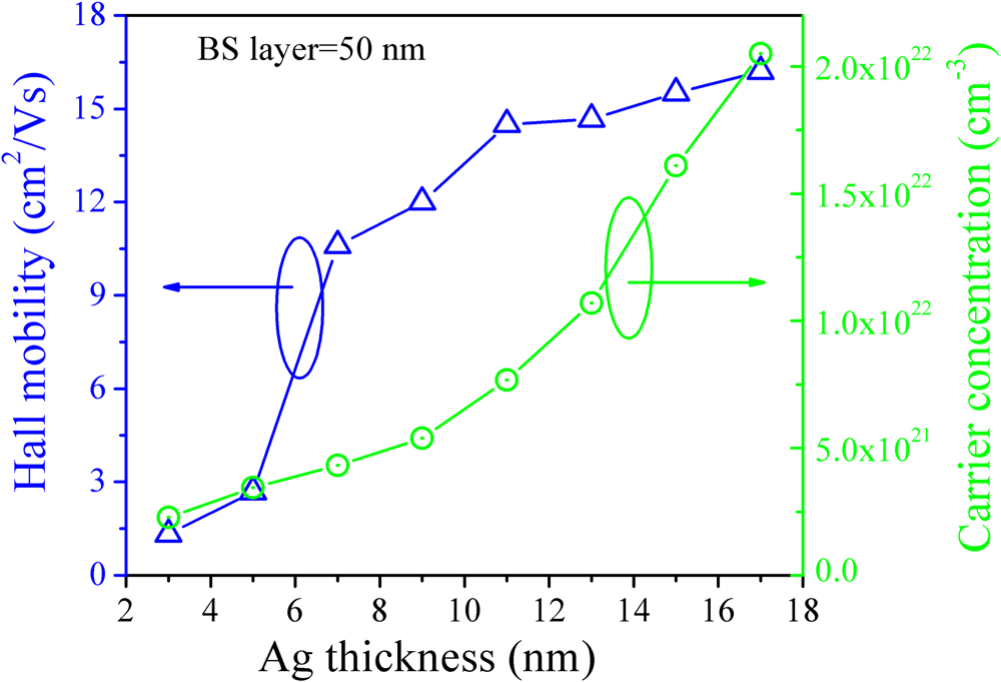
Hall mobility and carrier concentration of the BS/Ag/BS multilayer flexible thin films as a function of Ag thickness.

The resistivity can be explained using the following basic relation:

9 $${\rm{\rho }}=\frac{1}{ne\mu }$$

where *n* is the carrier concentration, *e* is the Charge of electron, and *μ* is the carrier mobility. The carrier concentration and mobility BS/Ag/BS multilayer flexible thin films with various Ag mid-layer thickness are shown in Fig. 3. The Hall mobility of multilayer flexible thin films with 3–5 nm thick Ag mid-layer is very low (<3.0 cm^2^/Vs). At this thickness regime, the low mobility is lead to the large amount of interface scattering (i.e., scattering of carriers at the Ag/BS and BS/Ag interfaces) due to the island structure of Ag mid-layer. As the thickness of Ag mid-layer increases, the Ag mid-layer becomes near-continuous or continuous, and then the interface scattering decreases. As a consequence, the mobility increases with the increase of the Ag mid-layer thickness. The increase in the carrier concentration with increasing Ag mid-layer thickness can be explained on the basis of Schottky theory. There is formation of an Ohmic contact at the Ag/BS interface due to the work function of BS is higher than that of Ag^32^. After the Ag contact with BS, an accumulation of electrons occur in the BS layer, and there is significant injection of electrons into the BS layer. The conduction and valence bands of BS curve downward due to these electrons transfer, until receiving a thermodynamic equilibrium, the Fermi level across the interface in a straight line at this time. In this case, the electron in the Ag mid-layer will flow into BS layer without barrier. Therefore, the carrier concentration in BS layer massive increases as the thickness of Ag mid-layer increases.

The figure of merit (FOM), as defined by Haacke^33^, is commonly used to reflect the trade–off between optical transmittance and electrical conduction. The *FOM* is estimated as follow^33^:

10 $$FOM=\frac{{T}_{av}^{10}}{{R}_{sh}}$$

**Figure 4:**
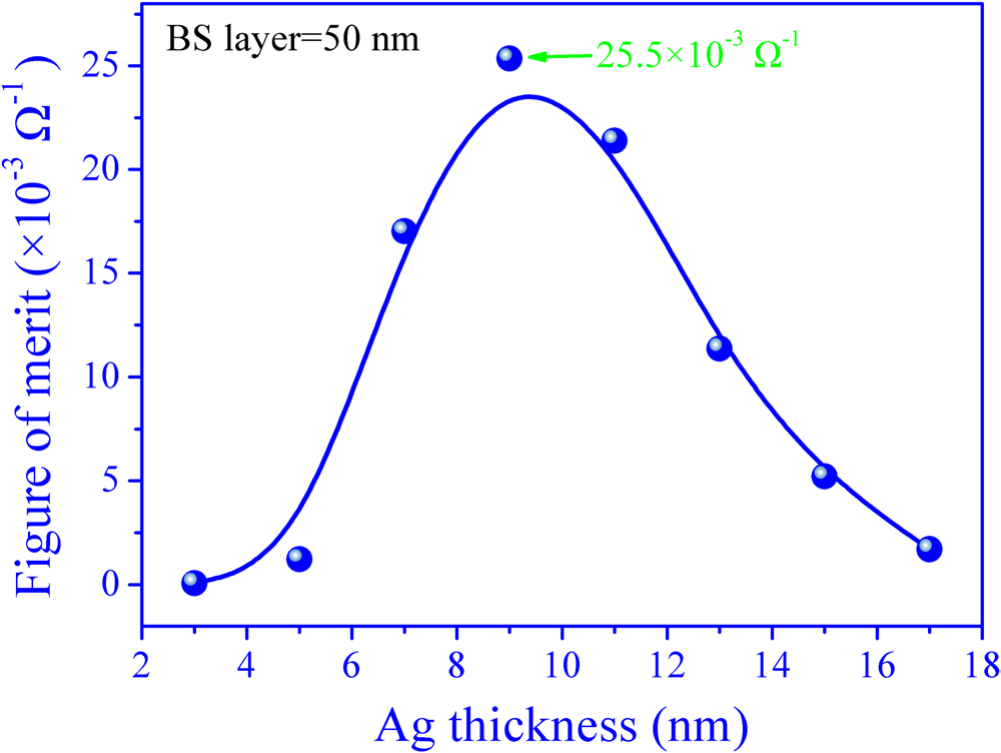
The *FOM* values of the BS/Ag/BS multilayer flexible thin films as a function of Ag thickness.

Figure 4 shows a plot of *FOM* for the for the BS/Ag/BS multilayer flexible thin films with diffident Ag mid-layer thickness. From the plot, it can be seen that the *FOM* value initially increases with thickness of Ag mid-layer, reaches a maximum (25.5 × 10^−3^ Ω^−1^) at 9 nm, and decreases with further increasing Ag mid-layer thickness to 17 nm. The largest *FOM* value is obtained when the thickness of Ag mid-layer is 9 nm.

### Effect of the BS layers thickness

According to the above results, we know that the BS/Ag/BS multilayer flexible thin films exhibit the largest *FOM* value when the thickness of Ag mid-layer is 9 nm. The optimal Ag thickness is invariable with varying the oxygen layer thickness according to Yu^14^, In order to investigate the role of BS layers in the multilayer flexible thin films, optical and electrical properties of the multilayer flexible thin films with various BS layers thicknesses and the fixed thickness of Ag mid–layer (9 nm) was investigated in the below sections.

**Figure 5:**
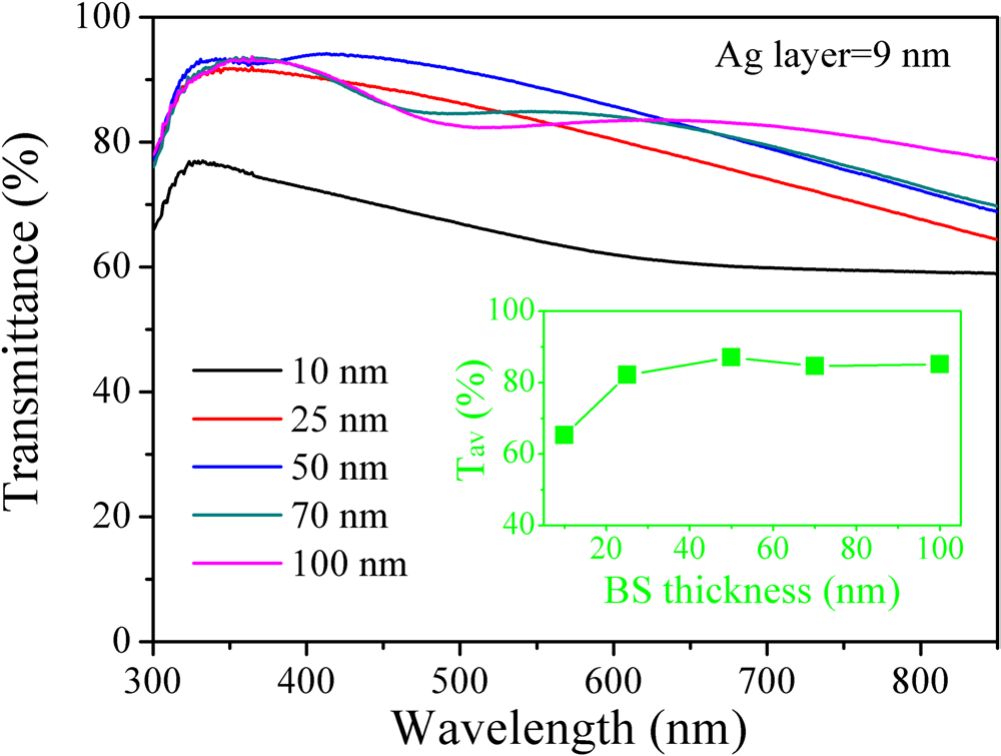
Transmittance spectra of BS/Ag (9 nm)/BS multilayer flexible thin films with various thicknesses of BS layers. Inset is the average optical transmittance in the visible range (380 nm~780 nm) of the multilayer flexible thin films as a function of BS layers thickness.

The optical transmittance spectra of BS/Ag (9 nm)/BS multilayer flexible thin films with different BS layers thicknesses is depicted in Fig. 5. The values of average optical transmittance in the visible range (380–780 nm) calculated from the Eq. (1) are shown in the inset of Fig. 5. The average optical transmittance of BS (10 nm)/Ag (9 nm)/BS (10 nm) multilayer flexible thin films is low because of the strong light reflecting form the Ag mid-layer and the PC substrate. The light scattering is caused by the near-continuous surface structure may be another factor influence the transmittance. With increasing the thickness of BS layers from 10 to 50 nm, the average optical transmittance initially increases, reaches a maximum (~87%), and slightly decreases with the thickness of BS layers further increase to 100 nm. According to Swanepoel^34^, the average refractive index of BS can be calculated by the follow equation:

11 $$r=\frac{1}{{T}_{av}}+{(\frac{1}{{T}_{av}^{2}}+1)}^{1/2}$$

where *r* is the average refractive index. The average refractive index of bare BS thin films are obtained according to Eq. (11) is ~1.38. The minimum thickness of the antireflective BS films can be determined as follow^34^:

12 $${d}_{m}=\frac{\lambda }{4r}$$

where *d*_*m*_ is the minimum thickness, *λ* is the optical wavelength (we use the wavelength of 550 nm which is much sensitive to human eyes). The minimum thickness BS films is calculated is ~100 nm. That is to say, the BS layers play a role in the antireflection layer to block the reflected light when the total thickness of BS layers is above 100 nm (the single layer thickness ≥50 nm). However, thicker films tend to absorb more light and degrade optical transparence^35^, therefore, the average optical transmittance decreases slightly with the thickness of BS layers further increase.

**Figure 6:**
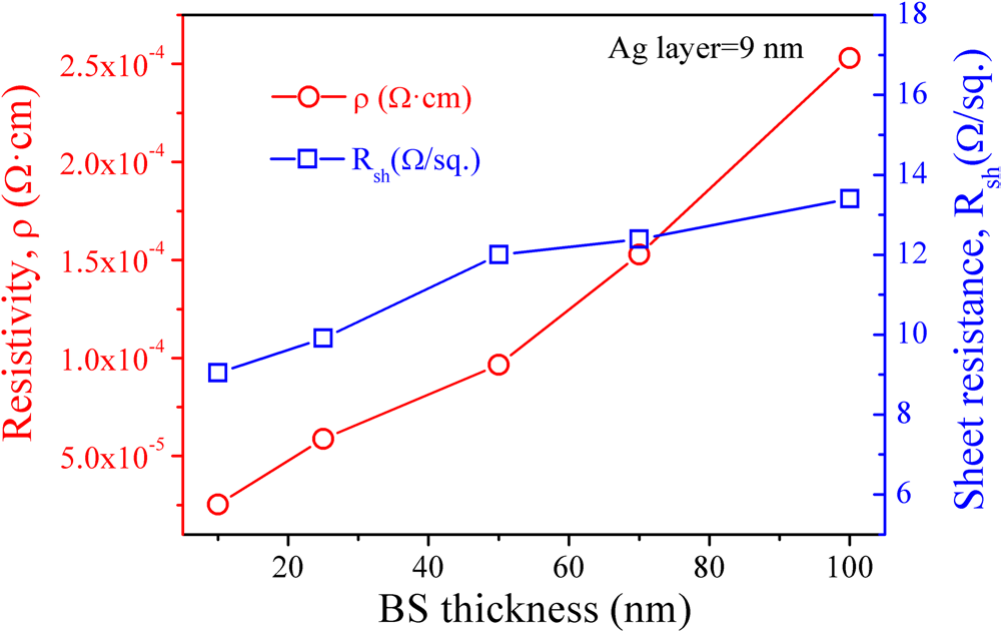
The dependence of resistivity and sheet resistance for BS/Ag (9 nm)/BS multilayer flexible thin films on the BS layers thickness.

Figure 6 presents the effect of BS layer thickness on the resistivity and sheet resistance of the BS/Ag(9 nm)/BS multilayer flexible thin films deposited at room temperature. When the thickness of BS layers is 10 nm, the resistivity and sheet resistance of multilayer flexible thin films are 2.53 × 10^−5^ Ω · cm and 9.04 Ω/sq., respectively. With increasing BS layers thickness from 10 nm to 100 nm, the resistivity increase linearly and the sheet resistance increases slowly. Since *ρ*_*Ag*_ ≪ *ρ*_*BS*_, Eq. (5) can be written as

13 $$\rho \approx \frac{(2{d}_{BS}+{d}_{Ag}){\rho }_{Ag}}{{d}_{Ag}}$$

**Figure 7:**
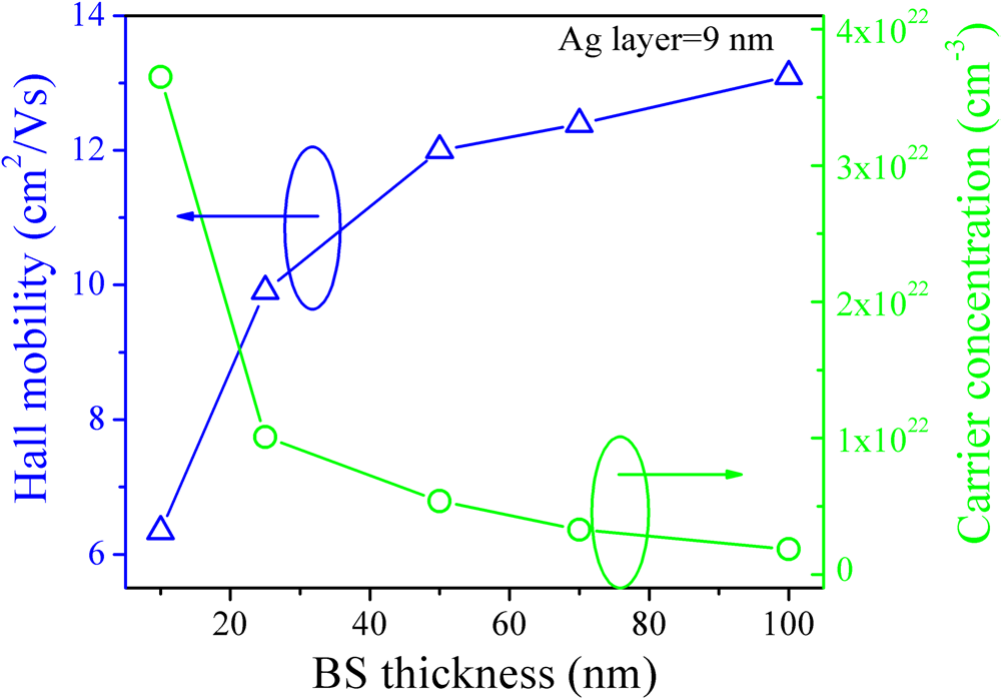
Hall mobility and carrier concentration of the BS/Ag (9 nm)/BS multilayer flexible thin films as a function of BS layers thickness.

It can be clearly seen that the resistivity increases as the increase of BS layers thickness. The detailed variation information of resistivity and sheet resistance can be understand by analyzing the carrier concentration and hall mobility. Figure 7 shows the carrier concentration and hall mobility of BS/Ag(9 nm)/BS multilayer flexible thin films as a function of BS layer thickness. The increased mobility is attributed to the improved film quality of BS layers that weakens defect scattering and increases carrier lifetime^36^. In the BS/Ag(9 nm)/BS multilayer flexible thin films, the almost all carriers are provided by the 9 nm thick Ag mid-layers, it should be obvious that the carrier concentration of BS/Ag(9 nm)/BS multilayer flexible thin films decreases as the increase of BS layers thickness.

The figures of merit, which are determined according to Eq. (3), are 1.2 × 10^−3^, 14.5 × 10^−3^, 25.5 × 10^−3^, 18.6 × 10^−3^ Ω^−1^ and 14.7 × 10^−3^ Ω^−1^ for the BS/Ag (9 nm)/BS multilayer flexible thin films with 10, 25, 50, 70 and 100 nm thick BS layers, respectively. The highest figure of merit (25.5 × 10^−3^) is obtained when the thickness of both top and bottom BS layers is 50 nm and the thickness of Ag mid–layer is 9 nm. The comparison of the best FOM values between the literature and the proposed structures references to the Table S2 of SI. The electrical stability of multilayer flexible thin films references to the Fig. S3 and Table S2 of SI.

## Conclusion

In conclusion, high conductivity BaSnO_3_/Ag/BaSnO_3_ multilayer flexible thin films with good transmittance have been obtained by magnetron sputtering at room temperature. The optical and electrical properties are strongly dependent on the thicknesses of Ag mid-layer and BaSnO_3_ layers. The resistivity decreases with the thickness of Ag mid–layer increases. The resistivity increases as the thicknesses of both top and bottom BaSnO_3_ layers increases. The figure of merit value of BaSnO_3_/Ag/BaSnO_3_ multilayer flexible thin films with optimal thicknesses of Ag mid-layer (9 nm) and BaSnO_3_ layers (50 nm), which has a resistivity of 9.66 × 10^−5^ Ω · cm, a sheet resistance of 9.89 Ω/sq. and an average transmittance of above 87% in the visible range from 380 to 780 nm, is 25.5 × 10^−3^ Ω^−1^. The change rate of resistivity is under 10% after repeated bending of the multilayer flexible thin films. These results indicate that BaSnO_3_/Ag/BaSnO_3_ multilayer flexible thin films, which are indium free, can be used as a high-performance flexible transparent electrodes in flexible optoelectronic devices such as flexible electronic circuits, flexible electronic book, organic photovoltaics, and flexible organic light emitting diodes.

## Methods

The BaSnO_3_/Ag/BaSnO_3_ multilayer thin films were prepared on PC substrates by RF magnetron sputtering of BaSnO_3_ using a BaSnO_3_ ceramic target and DC magnetron sputtering of Ag using a Ag target (99.99% purity, 60 mm diameter, 0.30 cm thickness) in an inline magnetron sputtering deposition system at room temperature. The PC substrates were ultrasonically cleaned in acetone for 30 min, rinsed in absolute ethyl alcohol and subsequently dried before the deposition. The target–to–substrate distance was 6 cm. Prior to sputtering, the vacuum chamber was evacuated to a base pressure of lower than 6.0 × 10^−4^ Pa. The working pressure for deposition was maintained at 0.5 Pa using high purity (99.999%) Ar ambient gas. Both the top and bottom BaSnO_3_ layers were deposited by RF magnetron sputtering at 50 w, the deposition rate was about 5 nm/min. Ag mid–layer was deposited by DC magnetron sputtering at 40 w, the deposition rate was 1 nm/s. The thickness of the Ag mid–layer was varied between 0 and 17 nm and the both the top and bottom BaSnO_3_ layers varied between 10 and 100 nm. The thickness of BaSnO_3_ layers and Ag mid–layers was estimated based on the deposition time and deposition rate.

### Characterization

The crystal structure of the films was characterized by X–ray diffraction using a (XRD DX–2500, FangYuan, PR China) system. The thickness of the thin films was measured by Alpha–Step D–100 profilometer (KLA–Tencor, California, USA). The electrical properties (electrical resistivity, Hall mobility, carrier concentration, and sheet resistance) were measured by Hall measurements in the van der Pauw configuration (Ecopia HMS 3000 Hall System, Republic of Korea) and four-point probe instrument (SX1934, SuZhou, PR China). Optical transmittance spectra and absorption spectra were obtained on an ultraviolet–visible–near infrared (UV–Vis–NIR) spectrophotometer (Varian Cary 5000, USA) in the wavelength range 300–850 nm.

## Electronic supplementary material

SUPPLEMENTARY INFO
