# Supplementary material for: High quality transparent conductive Ag-based barium stannate multilayer flexible thin films
Source: Sci Rep. 2017 Mar 7;7:103. doi: 10.1038/s41598-017-00178-9 (PMC5427918; doi:10.1038/s41598-017-00178-9)
Supplement: Supplementary file 1 — SUPPLEMENTARY INFO [file 41598_2017_178_MOESM1_ESM.doc]

**High quality transparent conductive Ag-based barium stannate multilayer flexible thin films**

**Muying Wua[[1]](#footnote-2)*, Shihui Yu^b^, Lin He^a^, Lei Yang^a**^, Weifeng Zhang^c^**

*^a^School of Electronic Engineering, Dongguan University of Technology, Guangdong Dongguan, 523808, China*

*^b^School of Electronic and Information Engineering, Tianjin University, Tianjin 300072, P. R. China*

*^c^Key Laboratory of Photovoltaic Materials of Henan Province and School of Physics and Electronics, Henan University, Kaifeng 475004, P. R. China*

***Effect of the Ag mid-layer thickness***

The XRD patterns of BS/Ag/BS multilayer flexible and bare BS thin films are exhibited in Figure S1. For the BS/Ag/BS multilayer flexible thin films, both the thicknesses of top and bottom BaSnO_3_ layers are 50 nm, the thickness of Ag mid-layer is 11 nm, the schematic of flexible multilayer structure is shown in inset of Figure. S1. The dispersing diffraction peak labeled “X” for the two kinds of thin films are x-ray peaks from the PC substrate. No peaks of metal Ag can be observed, it may be due to the layer was too thin, similar to what was previously reported by Li group.^1^ The crystal temperature of BaSnO_3_ thin films is above 700 °C, therefore, the BaSnO_3_ layers of the multilayer flexible thin films deposited at room temperature are amorphous and no peaks of BaSnO_3_ could be detected in the XRD patterns.

In order to intuitive observation the surface morphology of BS/Ag/BS multilayer flexible and bare BS thin films, the AFM was used. Figure S2 shows the AFM micrographs of multilayer flexible and bare BS thin films. As shown in Figure S2a, for bare BS thin films, the surface morphology is a compact structure. For the multilayer flexible thin films with Ag thickness of 3 nm, as shown in Figure S2b, the surface morphology is an incompact structure, the nucleation process has been completed and nucleus growth has already proceeded and island structure appeared. When the Ag thickness increased to 9 nm (as shown in Figure S2c), the islands connected to each other and a coalescence phenomenon with channel was observed. For the film with the Ag mid-layer thickness of 17 nm, some grains are formed and uniformly distributed on the surface.

Both the electrical and optical properties are very important for the applications of flexible transparent conductive films. Ideally, both optical transmittance and electrical conduction should be as large as possible. However, their interrelation excludes the simultaneous achievement of maximum transmittance and conduction in most cases. Obtaining the material for the best performance is based on the optimal tradeoff between the optical transmittance and electrical conduction. The higher the FOM value, the better the optical and electrical properties. The comparison of the best FOM values between the literature and the proposed structures is summarized in Table S1. The FOM value of BS/Ag (9 nm)/BS multilayer flexible thin films prepared in this paper is much higher than that of the other TCO/metal/TCO multilayer system or single TCOs, suggesting that BS/Ag (9 nm)/BS multilayer flexible thin films have the excellent optical and electrical properties, which is advantages for application.

***Electrical stability of multilayer flexible thin films***

All of the measurements were carried out at room temperature. The electrical stability was evaluated based on changes in the s resistivity after bending the multilayer flexible thin films around a cylinder with a radius of 15 mm.

The electrical stability of the BS/Ag/BS multilayer flexible thin films was evaluated based on the changes in the resistivity after bending the stack around a cylinder. The multilayer flexible thin films sheet were bent into a curve with a surface curvature radius (R) of 15 mm, as shown in Figure S3. The BS (50 nm)/Ag (9 nm)/BS (50 nm) multilayer flexible thin film with the highest figures of merit were consisted as the bending test sample. The changes of electrical properties (resistivity, carrier concentration and hall mobility) as a function of bending times are shown in Table S2. The resistivity slightly increases as the increase of bending times. After 50 bending cycles, the resistivity does not show noticeable variation, it only increases by 9% compared with the as-deposited multilayer flexible thin films. Under mechanical stress, imperfections and micro-cracks are formed influencing the consecutiveness of Ag mid-layer and leading to the slight reduction of carrier concentration, therefore the resistivity slightly increases.

**References**

1. Yu S. *et al.* Optimization of SnO_2_/Ag/SnO_2_ tri–layer films as transparent composite electrode with high figure of merit. *Thin Solid Films* **552**, 150–154 (2014).
2. Dimopoulos T. *et al.* Properties of transparent and conductive Al: ZnO/Au/Al: ZnO multilayers on flexible PET substrates. *Mater. Sci. Eng. B* **200**, 84-92 (2015).
3. Huang Q. *et al.* Highly thermostable, flexible, transparent, and conductive films on polyimide substrate with an AZO/AgNW/AZO structure. *ACS Appl. Mater. Interfaces* 2015, **7**, 4299−4305 (2015).
4. J. Han, S. Yuan, L. Liu, X. Qiu, H. Gong, X. Yang, C. Li, Y. Hao and B. Cao, *J. Mater. Chem. A* 2015, **3**, 5375–5384
5. Han J. *et al.* Fully indium-free flexible Ag nanowires/ZnO: F composite transparent conductive electrodes with high haze. *J. Mater. Chem. C*, **2**, 3750–3755 (2014).
6. Dhar A. & Alford. T. L. Optimization of TiO_2_/Cu/TiO_2_ multilayer as transparent composite electrode (TCE) deposited on flexible substrate at room temperature. *ECS Solid State Lett.* **3**, N33–N36 (2014).
7. Miao D. Jiang S. Shang S. & Chen Z. Highly transparent and infrared reflective AZO/Ag/AZO multilayer film prepared on PET substrate by RF magnetron sputtering. Chen, *Vacuum*, **106**, 1–4 (2014).
8. Abachi T. *et al.* Highly flexible, conductive and transparent MoO_3_/Ag/MoO_3_ multilayer electrode for organic photovoltaic cells. *Thin Solid Films* **545**, 438–444 (2013).
9. Park S. U. & Koh J. H. Low temperature rf-sputtered In and Al co-doped ZnO thin films deposited on flexible PET substrate. *Ceram. Inter.* **40**, 10021–10025 (2014).
10. Kim H. Horwitz J. S. Kushto G. P. Kafafi Z. H. & Chrisey D. B. Indium tin oxide thin films grown on flexible plastic substrates by pulsed-laser deposition for organic light-emitting diodes. *Appl. Phys. Lett.* **79**, 284 (2001).
11. Huang, X. *et al.* Preparation of fluorine-doped tin oxide (SnO_2_: F) film on polyethylene terephthalate (PET) substrate. *Mater. Lett.*, **64**, 1701–1703 (2010).
12. Jeong J. A. Shin H. S. Choi K. H. & Kim H. K. Flexible Al-doped ZnO films grown on PET substrates using linear facing target sputtering for flexible OLEDs. *J. Phys. D: Appl. Phys.* **43**, 465403 (2010).

**Supplementary Figure S1**

**
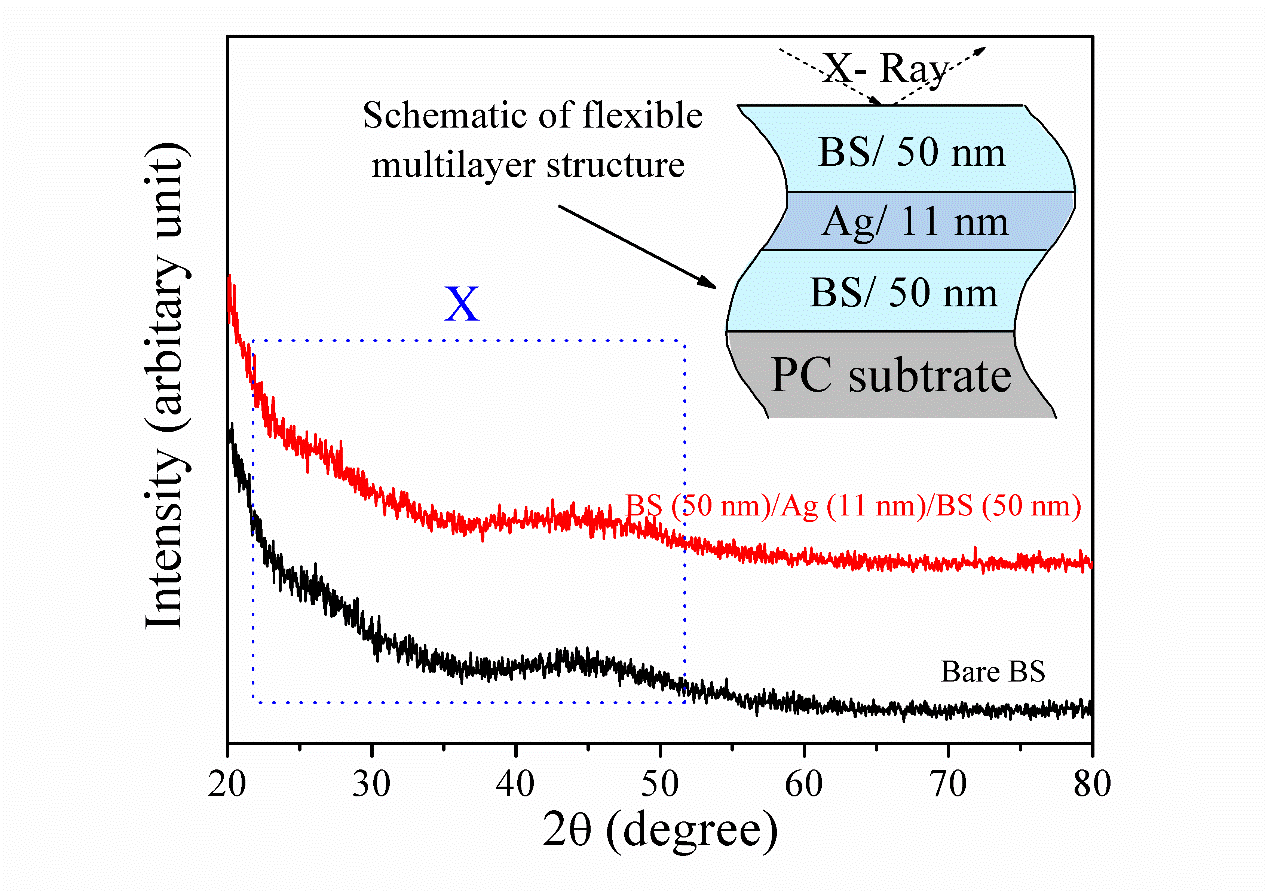
**

**Figure 1**. XRD patterns of BS thin films and the BS/Ag/BS multilayer flexible thin films with 11 nm thick Ag mid-layer. Inset is the schematic of flexible multilayer structure.

**Supplementary Figure S2**


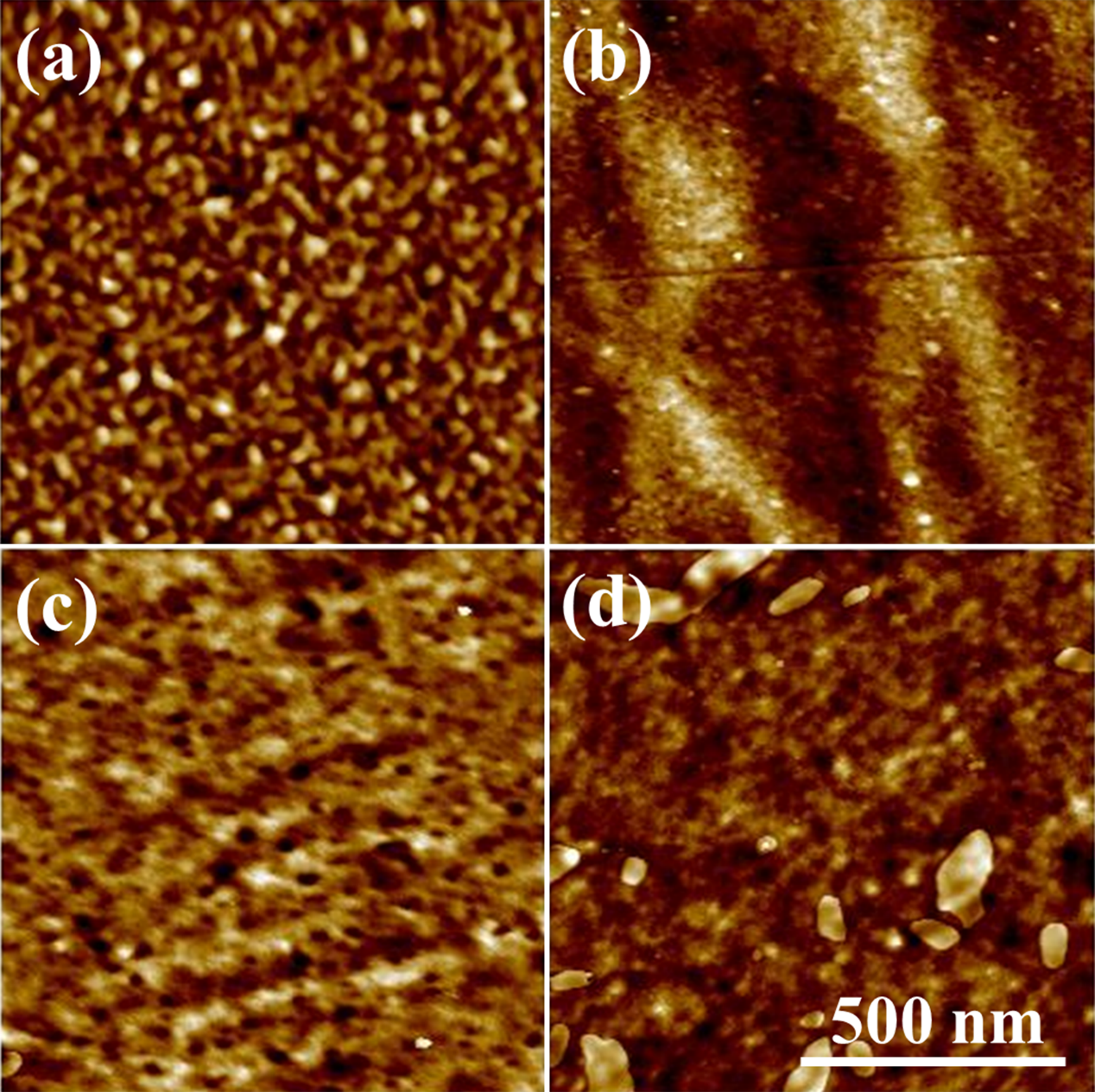


**Figure S2**. AFM micrographs of multilayer flexible with various Ag mid–layer thicknesses: (a) 0 nm, (b) 3 nm, (c) 9 nm, (d) 17 nm.

**Supplementary Figure S3**


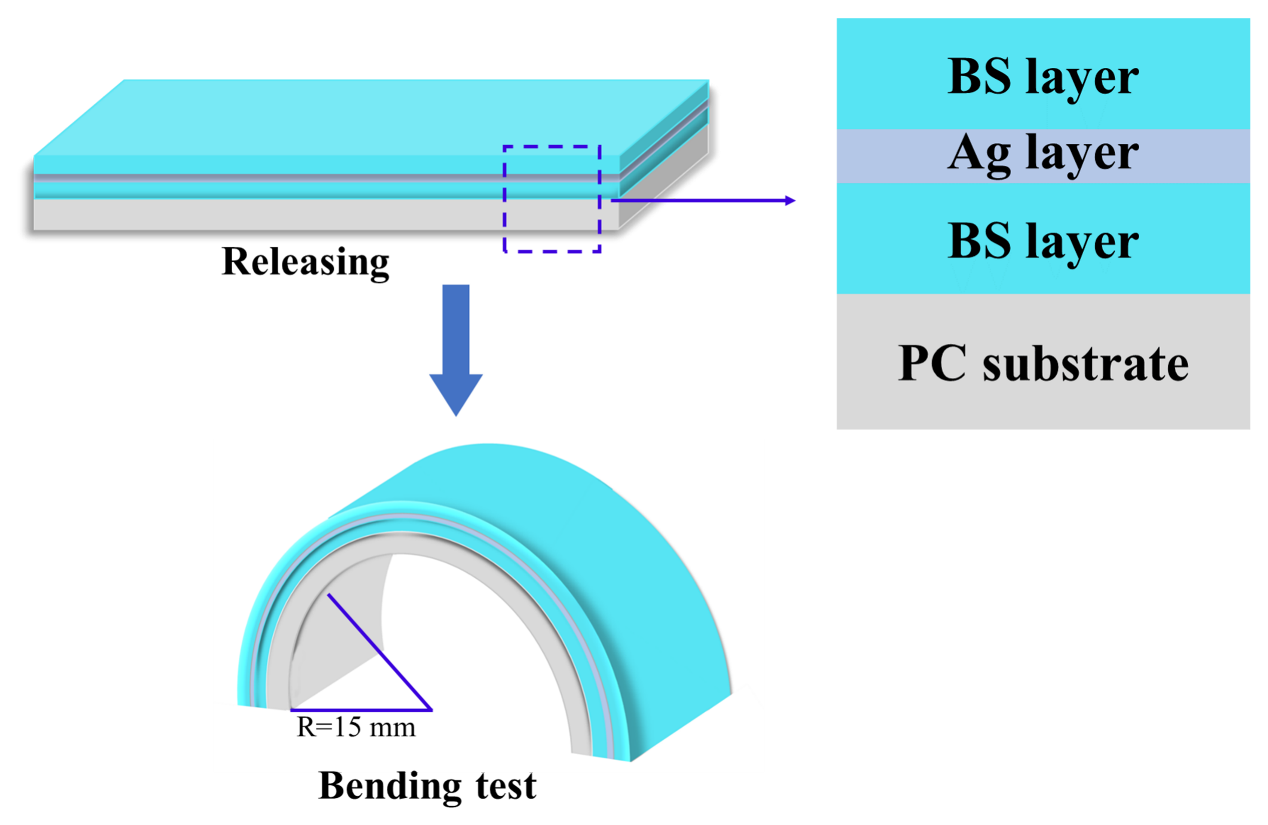


**Figure S3**. schematic of the flexible evaluation by applying a bending radius R for the BS (50 nm)/Ag (9 nm)/BS (50 nm) multilayer flexible thin films.

**Supplementary Table S1**

| **Ref.** | **Process method** | **Structure** | **Thickness**  **(nm)** | **Transmittance**  **(%)** | **sheet resistance**  **(Ω/sq.)** | **FOM**  **(×10^−2^ Ω^−1^)** |
| --- | --- | --- | --- | --- | --- | --- |
| **[2]** | Sputtering | AZO/Au/AZO  (PET substrate) | (50/11/50) | ~80  (390-700 nm) | ~26 | 0.4 |
| **[3]** | Sputtering. | AZO/AgNW/AZO  (PI substrate) |  | 74.4  (550 nm) | 8.6 | 0.6 |
| **[4]** | PLD | AgNW/FZO  (PET substrate) |  | 83  (550 nm) | 17 | 0.91 |
| **[5]** | Sputtering | AZO/AgNW/AZO  (PET substrate) |  | 80.5  (550 nm) | 27.6 | 0.41 |
| **[6]** | Sputtering | TiO_2_/Cu/TiO_2_  (PEN substrate) | (30/6/30) | 81  (380-780 nm) | 19 | 0.64 |
| **[7]** | Sputtering | AZO/Ag/AZO  (PET substrate) | (30/10/30) | 78.5  (400-700 nm) | 10.19, | 0.87 |
| **[8]** | Vacuum Evaporation | MoO_3_/Ag/MoO_3_  (PET substrate) | (17.5/11/17.5) | 74  (300-700 nm) | 13 | 0.42 |
| **[9-12]** |  | Single TCOs, such as ITO, AZO, FTO, ect  (flexible substrate) |  | (>80)  (380-780 nm) | ~100 | ~0.1 |
| This study | Sputtering | BS/Ag/BS | (50/9/50) | 87  (380-380 nm) | 9.89 | 2.55 |

Table S1. Comparison of the best figure of merit between the literature and the proposed structures.

AZO: Al doped ZnO; FTO: F doped SnO_2_; AgNW: Ag nanowire; PET: polyethylene terephthalate; PI: polyimide; PEN: polyethylene naphthalate.

**Supplementary Table S2**

Table S2 The dependence of electrical properties (resistivity, hall mobility and Carrier concentration) of BS (50 nm)/Ag (9 nm)/BS (50 nm) multilayer flexible thin films as a function of bend cycle with a curvature radius of 15 mm..

| **Bend-cycles** | **Resistivity**  (×10^-5^ Ω∙cm) | **Hall mobility**  (cm^2^/Vs) | **Carrier concentration**  (×10^21^ cm^-3^) |
| --- | --- | --- | --- |
| 0 | 9.66 | 12.00 | 5.38 |
| 5 | 9.70 | 11.98 | 5.37 |
| 10 | 9.69 | 12.08 | 5.31 |
| 20 | 9.85 | 11.92 | 5.28 |
| 30 | 10.14 | 12.10 | 5.03 |
| 40 | 10.35 | 11.89 | 4.94 |
| 50 | 10.51 | 12.28 | 4.76 |

1. ^^ Corresponding author. Tel.: +86 769 228611.

   E-mail address: [*wumy@dgut.edu.cn*](mailto:wumy@dgut.edu.cn)*, wumy01@126.com* (M. Wu).

   ^**^Corresponding author. Tel.: +86 769 228611.

   E-mail address: [*yangl@dgut.edu.cn*](mailto:yangl@dgut.edu.cn) (L. Yang). [↑](#footnote-ref-2)
